# Assessing Mental Health and Emotional States by Using Smartphone Photoplethysmography–Based Digital Pulse Waveform Analysis: Cross-Sectional Observational Study

**DOI:** 10.2196/81301

**Published:** 2026-03-16

**Authors:** Ivan Liu, Luming Hu, Jing Luo, Chang Liu, Qi Zhong, Shiguang Ni

**Affiliations:** 1 Bay Area School of Applied Psychological Sciences Beijing Normal University Zhuhai, Guangdong China; 2 Department of Psychology Faculty of Arts and Sciences Beijing Normal University Zhuhai, Guangdong China; 3 Faculty of Psychology Beijing Normal University Beijing China; 4 Shenzhen International Graduate School Tsinghua University Shenzhen, Guangdong China

**Keywords:** mental health, mental well-being, happiness, smartphone photoplethysmography, digital pulse waveform, multimodal emotion recognition, affective computing, atherosclerosis, blood pressure

## Abstract

**Background:**

Pulse characteristics are well-established biomarkers of physical health; however, their relevance to psychological well-being remains insufficiently explored. A key barrier is the difficulty of acquiring pulse recordings and blood pressure measurements of adequate quality outside clinical or laboratory settings by using accessible measurement approaches.

**Objective:**

This study aimed to examine the feasibility of using smartphone photoplethysmography to extract fingertip pulse-waveform features and to evaluate their associations with psychological measures. It further aimed to systematically compare time-, curvature-, and frequency-domain pulse-waveform features in relation to psychological variables.

**Methods:**

A total of 127 students and university employees in Shenzhen, China, were recruited. Participants recorded repeated 4-minute fingertip videos by using a custom smartphone app while a fingertip oximeter simultaneously acquired reference pulse signals. Smartphone videos were converted into photoplethysmography signals, segmented into beat-to-beat intervals, and summarized into time-, curvature-, and frequency-domain features, with normalization for heart rate and stature. Psychological well-being and mental health were assessed using the Satisfaction With Life Scale, Subjective Vitality Scale, Positive and Negative Affect Schedule, Patient Health Questionnaire-9, Generalized Anxiety Disorder-7, and the Self-Assessment Manikin. Associations between pulse-waveform features and psychological measures were examined using univariate regression with participant-level aggregation and cluster-robust standard errors. Random forest models evaluated multivariate predictive performance by using participant-level cross-validation. Agreement between smartphone-derived and oximeter-derived waveform features was assessed using Bland–Altman analysis.

**Results:**

Correlation analyses revealed strong within-domain associations among time-, curvature-, and frequency-domain pulse-waveform features, with comparatively weaker cross-domain correlations. A correlation-based feature-selection procedure reduced multicollinearity and yielded a final set of 7 features: estimated reflection index, crest time (CT), the third curvature minimum (F/A), the fourth curvature minimum (H/A), the first power spectrum density component, the baseline of Fourier decomposition (V0), and systolic blood pressure. Univariate regression analyses indicated that negative psychological states were primarily associated with time- and curvature-domain features. Depressive symptoms were significantly related to F/A, V0, and the estimated reflection index. Anxiety showed an association with F/A, and negative affect was associated with CT and F/A. In contrast, positive affect measures showed fewer and weaker associations. Valence was related to F/A and H/A, whereas arousal was associated with CT and H/A. Random forest models demonstrated statistically significant but modest predictive performance for negative mental health outcomes, with weaker performance for positive affect. Bland–Altman analyses indicated minimal systematic bias for outcomes with significant predictive correlations. Comparisons with an oximeter showed significant correlations and acceptable agreement, with time-domain features demonstrating greater robustness than reflection-based metrics.

**Conclusions:**

Smartphone-based photoplethysmography can capture pulse-waveform features associated with psychological measures, particularly negative psychological states. However, predictive performance remains limited, and variability in signal quality from user-operated recordings poses a practical challenge.

## Introduction

Assessing physiological health by monitoring pulse rhythm and waveforms has been practiced for millennia [1,2]. Through the analysis of pulse-waveform contours, clinicians gain insight into circulatory status and related physiological influences [3]. Numerous studies have associated waveform characteristics with factors including height, ethnicity [4], sex [5], respiration [6], and autonomic nervous system activity [7], and have used these features to predict medical conditions such as hypertension [8], heart failure [9], lipid metabolism disorders [3], renal disease [10], and diabetes mellitus [11].

**Figure 1:**
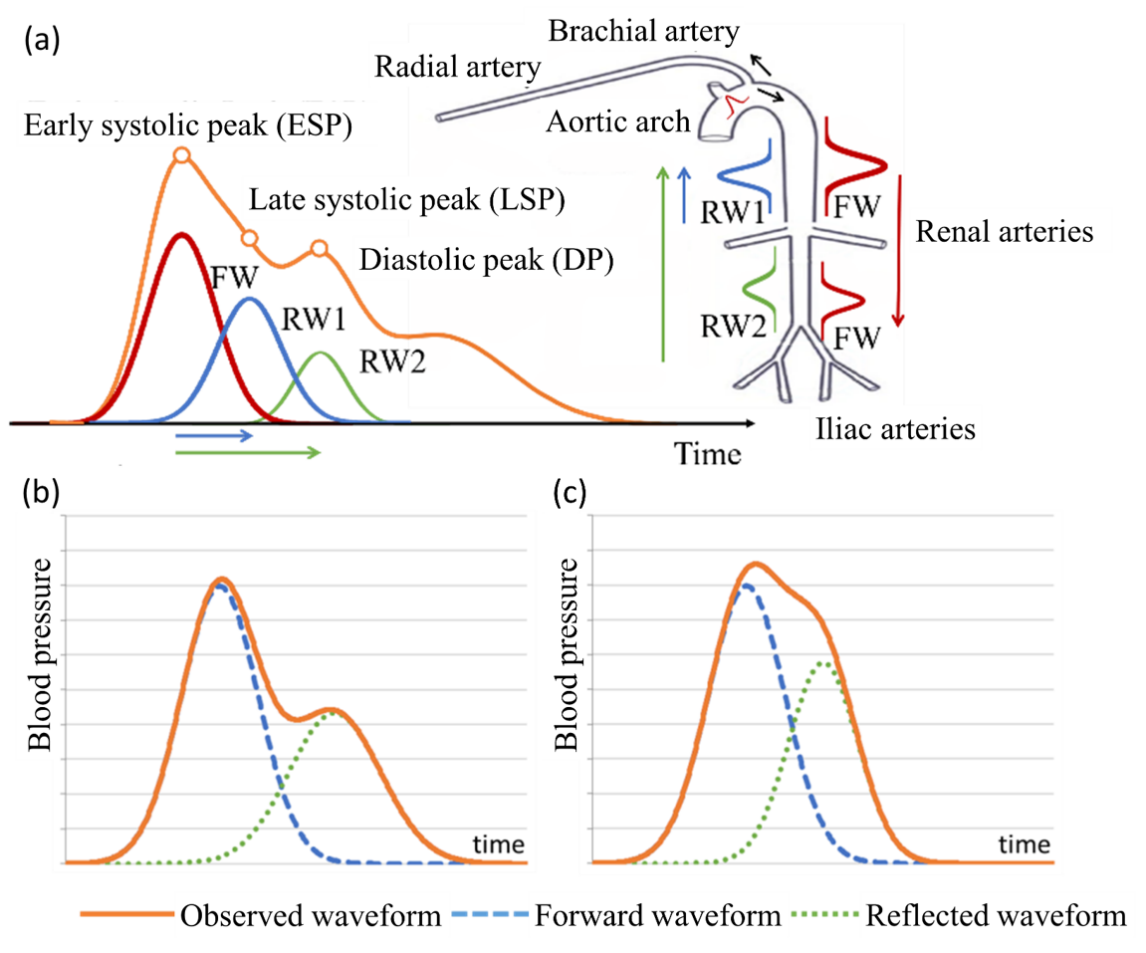
(A) The reflected waves and the shape of the radial arterial pulse waveform (reproduced from Liu et al [13], which is published under Creative Commons Attribution-NonCommercial-NoDerivatives 4.0 International License [17]). (B) An illustration of the radial arterial pulse waveform of healthy young adults (adapted from Baruch et al [8], which is published under Creative Commons Attribution 2.0 Generic License [18]). (C) The pulse waveform of people with signs of arterial aging. The reflected wave arrives earlier, boosts the systolic blood pressure, and changes the pulse contour [12,19]. FW: forward wave; RW: reflected wave.

The relationship between pulse waveforms and various health conditions can be explained by arterial stiffening. During ventricular systole, the left ventricle ejects oxygenated blood into the aorta, generating a forward pressure wave that travels through the arterial tree. The recorded pulse waveform reflects the superposition of this forward wave and the wave reflected from peripheral arterial sites [12] (Figure 1A) [8,13]. In healthy arteries, the compliant arterial walls transmit pulse energy more slowly, causing the reflected waves to arrive later, producing a waveform characterized by a pronounced systolic peak and a well-defined dicrotic notch (Figure 1B). With aging or pathological stiffening, often initiated by endothelial injury and atherosclerotic remodeling [14], arterial compliance decreases, causing the reflected wave to return earlier, which augments the forward wave, raises systolic pressure, and blunts the dicrotic notch [15] (Figure 1C). Thus, specific modifications in waveform morphology serve as indicators of cardiovascular aging and related pathologies [16].

Because enduring psychological states and personality traits modulate physiological regulatory systems, particularly inflammatory pathways [20] and sympathetic nervous system activation [21], it is reasonable to assume that psychological factors also influence vascular waveforms. Empirical evidence has supported the assumption that individuals with psychological conditions, such as chronic stress [22], depression [23], bipolar disorder [24], anxiety [25], and panic disorder [26], frequently exhibit arterial stiffening. Consequently, maladaptive psychological states may be detectable via pulse waveform analysis. In support of this premise, early investigations have reported significant associations between pulse waveform characteristics and mental health issues, including depression [27], anxiety [18,28], affective states [29], chronic fatigue [30], and cognitive stress [31,32].

Although early studies have linked pulse waveform morphology to mental health, real-world applications remain limited. A key challenge lies in the cost and complexity of acquiring waveform features outside clinical environments. Most prior research has focused on aortic waveforms, obtained using invasive equipment and specialized personnel in hospital settings [33], thereby limiting scalability. More recently, a small number of pioneering studies have used photoplethysmography to capture peripheral arterial signals (eg, from radial or digital arteries) [34,35]. However, despite its lower technical requirements, photoplethysmography-based pulse analysis still typically relies on dedicated medical devices [36], which constrains its accessibility and widespread deployment.

To bridge the gap in the literature, this study has two primary objectives:

First, it proposes and validates an innovative technique for extracting fingertip (digital artery) pulse-waveform features pertinent to psychological evaluation using only a standard smartphone. Over the past decade, smartphone camera–based acquisition of cardiac signals has garnered substantial attention, and numerous investigations have demonstrated that heart rate (HR) and heart rate variability (HRV) derived from smartphone cameras can reflect alterations in psychological states [37]. This approach uses the phone’s built-in camera to record subtle, cardiac-induced skin color fluctuations at the fingertip and converts them into radial artery waveforms via photoplethysmography. However, the capacity of this technology to capture detailed pulse waveform morphology and its application to mental health assessment remains underexplored.

Second, existing studies have typically examined a limited number of pulse-waveform features in isolation, and comprehensive comparisons, particularly for digital pulse-waveform features, are largely lacking. Although prior research has explored associations between pulse-waveform characteristics and psychological states, it has focused almost exclusively on aortic indices, such as the augmentation index [38]. Therefore, evidence linking peripheral waveforms (eg, radial or digital artery signals) to mental health outcomes remains sparse. Given that peripheral waveform morphology differs substantially from aortic contours due to local anatomical and physiological factors [16], these signals warrant independent and systematic investigation. On the other hand, studies of radial or digital artery morphology have generally considered only a narrow subset of available features. To address these gaps, this study conducts an extensive examination of time-domain, frequency-domain, and curvature-domain pulse-waveform features, evaluates their interrelationships, and investigates their associations with psychological health.

## Methods

### Participant Recruitment

This study recruited a total of 127 participants, comprising university students and staff members, from the last author’s affiliated institution in Shenzhen, China (72/127, 56.69% male; mean age 22.78, SD 1.97 years). Participants were recruited through on-campus posters and electronic posters distributed via student social media platforms. Eligibility was restricted to university students and staff, ensuring that all participants were at least 18 years of age and had access to university facilities. Participants were required to have no clinically diagnosed mental disorders or cardiovascular conditions. No additional exclusion criteria were applied.

### Study Procedures and Data Collection

During data collection, participants were instructed to rest their arm on a table, hold a Mi 8 SE smartphone (Xiaomi) in their left hand, and place their index finger over the rear camera. Using the Heartily Happy app, developed by our team and publicly available on Google Play at the time of the study, participants recorded 7 fingertip videos, each lasting 4 minutes, at a resolution of 120×160 pixels. To further evaluate the accuracy and practical applicability of the proposed approach, participants were additionally instructed to place the index finger of their right hand on a US Food and Drug Administration–cleared fingertip oximeter (FDA 510(k): K082641; CMS50D, Contec Medical Systems Co Ltd) to simultaneously record pulse waveforms. This study is part of a broader research program examining the relationship between pulse waveforms and mental health. The same dataset has also been used in studies investigating blood pressure [13] and HRV [37].

### Signal Processing

**Figure 2:**
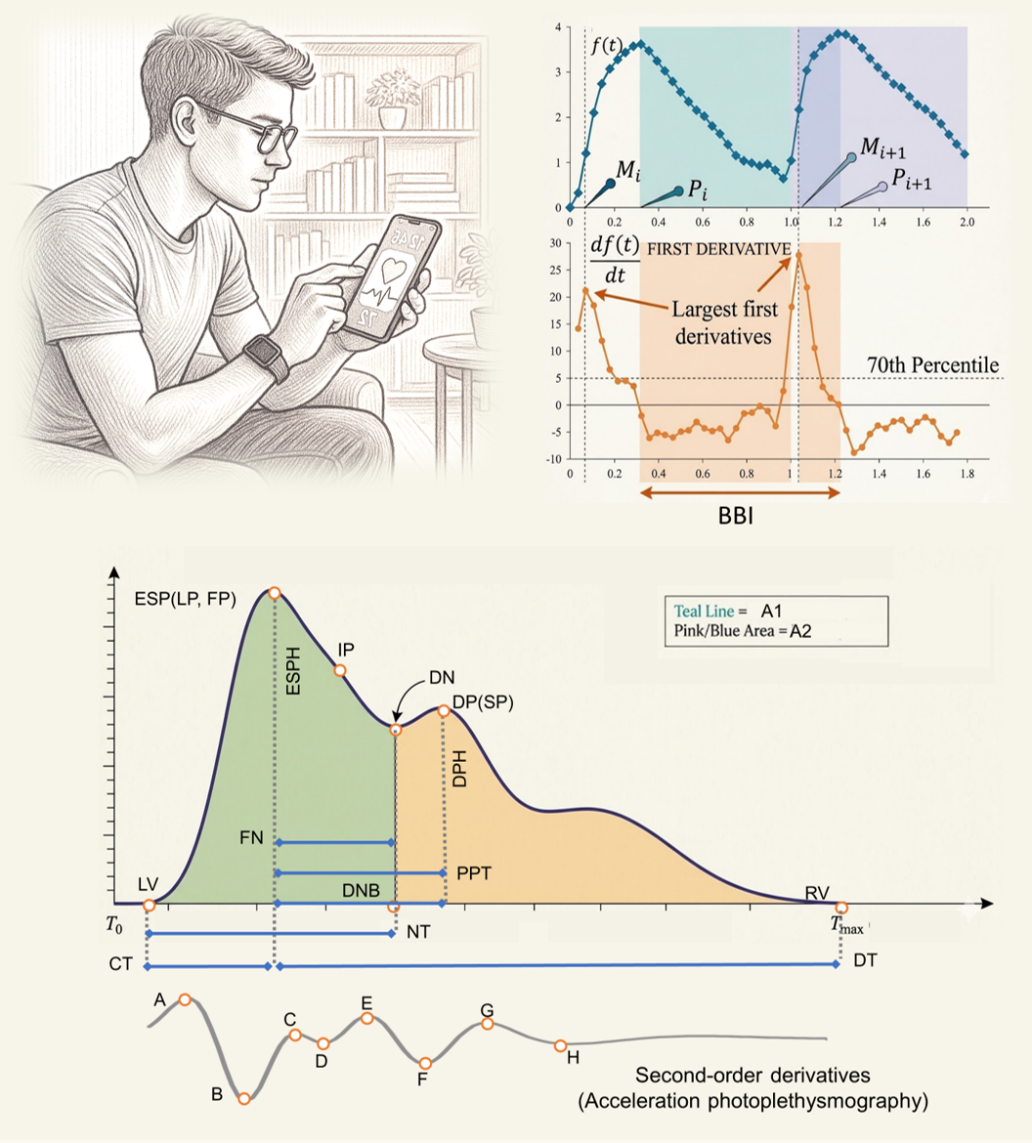
Smartphone photoplethysmography data collection and feature extraction. BBI: beat-to-beat interval; CT: crest time; DN: dicrotic notch; DP: diastolic peak; DPH: diastolic peak height; DT: diastolic time; ESP: the early systolic left peak; ESPH: early systolic peak height; FN: the interval between first peak to notch; FP: first peak; IP: inflection point; LP: left peak; LV: left valley; NT: notch time; PPG: photoplethysmography; PPT: peak-to-peak time; RV: reflected wave; SP: second peak.

The Heartily Happy app used the smartphone’s built-in flashlight to illuminate the fingertip and captured video at approximately 30 frames per second (Figure 2) [13,39]. Raw video frames, initially recorded in YUV format, were converted to red green blue color model for subsequent analysis. Waveform signals were extracted by computing a SD–weighted average across the 3 color channels.

Following peak detection, both photoplethysmography and oximetry signals were segmented into beat-to-beat intervals (BBIs). Waveform features were calculated for each BBI, and the median value of each feature across all BBIs was used to represent the sample. Detailed signal-processing procedures are described in [13,39].

Because pulse waveform length is inversely related to HR, following a conventional, although controversial, practice in aortic blood pressure research, waveform features were normalized to a reference HR of 75 beats per minute to control for HR-related effects [40-42]. In addition, since the timing of reflected waves depends on vascular path length, which is associated with body height, waveform features are often further normalized by stature; for example, the stiffness index is defined as body height divided by peak-to-peak time (PPT) [43]. Therefore, in this study, waveform features were normalized to a reference height of 170 cm to reduce stature-related interindividual variability. Sex was not included as a separate normalization factor, as its effects on waveform morphology are largely mediated by differences in body height.

### Waveform Metrics

**Figure 3:**
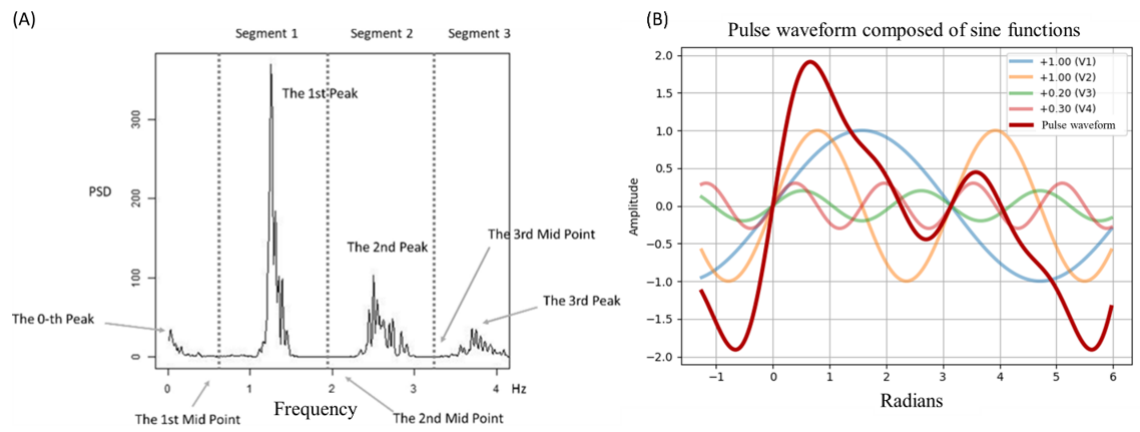
(A) An illustration of the power spectrum density generated by fast Fourier transform (reproduced from Liu et al [13], which is published under Creative Commons Attribution-NonCommercial-NoDerivatives 4.0 International License [17]). (B) An illustration of pulse waveform segmentation using sinusoidal functions.

This study considers 3 categories of waveform features commonly cited in pulse waveform analysis but underexplored in psychological research (Table 1 [13]). Time-domain indicators encompass measures of intervals between characteristic waveform points, amplitude differences among these points, and the total area under the waveform curve. Curvature-domain features are derived from the second derivative of the photoplethysmography waveform, often termed acceleration photoplethysmography, and quantify waveform contour and curvature [44]. Frequency-domain features are obtained via Fourier transform decomposition, with power spectral density functions characterizing the strength of each frequency component (Figure 3) [13].

**Table 1:** Waveform features (adapted from Liu et al [13]).

Name			Definition and calculation details
**Time-domain features**			
	SI^a^	The ratio of body height to PPT^b^ [45,46].	
	PPT	The interval between the ESP^c^, (also known as the LP^d^ or the FP^e^) and the DP^f^. The RV^g^ is the lowest point in the BBI^h^, and the preceding BBI’s RV is termed the LV^i^. The DP or SP is the first point between ESP and RV with a zero first derivative and a negative second derivative. If no second peak exists between ESP and RV, indicating a consistently negative slope from LP to RV, the DP is identified as the point with the minimum second derivative between ESP and RV.	
	EPPT	Estimated PPT	
	RI^j^	The ratio of the DPH^k^ to the estimated ESPH^l^ is a critical measure in this analysis.	
	ERI^m^	Estimated RI. Since the initial findings indicated that the auto-exposure adjustment feature of smartphone cameras could result in waveforms that appear smoother than those predicted by theoretical models, this study incorporates the ERI, as proposed by [13]. The ERI, defined as the ratio between the second peak and the estimated early systolic peak, serves as a substitute for the widely used Reflection Index to account for these discrepancies.	
	ESI^n^	Estimated SI	
	CT^o^	The interval between the LV and ESP [47].	
	NT^p^	The IP^q^ is defined as the last point before the second peak where the first derivative shifts from positive to negative. The lowest point between the end-systolic point (ESP) and the dicrotic peak DP^r^ is called the DN^s^. NT is the interval from the LV to the DN. In cases where there is no obvious second peak, the IP is designated as the DN.	
	IPP^t^	The percentage of BBIs in a sample where there is no obvious second peak and the IP is designated as the DN.	
	DT^u^	The interval between ESP and the RV [48]	
	IS^v^	The slope of the curve at the inflection point.	
	RCA	The ratio of CT to NT.	
	RDA	The ratio of NT to the combined duration of CT and DT.	
	FN	The interval between the first peak to notch.	
	SR	The interval between the second peak to RV	
	NV	The interval between the notch to RV	
	NH	Notch height.	
	SPH	Second peak height.	
	A1	The area under the curve from LV to the DN [32,49,50]. When there is no obvious second peak, IP is used as a surrogate for the DN. Since calculating the actual area involves more complex methods, this study approximates A1 as the polygon formed by LV, ESP, DN, and the DNB.	
	A2	The area under the curve from DN to RV [49]. This study approximates A2 as the triangle comprising IP, DN, and RV.	
	Inflection point area (IPA)	A2/A1 [49].	
**Curvature-domain features (acceleration** **photoplethysmography** **)**			
	A	The first local maximum of the second derivative is determined by the point with the largest second derivative before ESP.	
	B	The first local minimum of the second derivative, determined by the point with the smallest second derivative before ESP.	
	C	The second local maximum of the second derivative is determined by the point with the largest second derivative, point B, and point E.	
	D	The second local minimum of the second derivative is determined by the point with the smallest second derivative between points B and point E.	
	E	The third local maximum of the second derivative is determined by the point with the largest second derivative between ESP and point F.	
	F	The third local minimum of the second derivative is determined by the point with the smallest second derivative between the ESP and RV.	
	G	The fourth local maximum of the second derivative is determined by the point with the largest second derivative between point F and point H.	
	H	The fourth local minimum of the second derivative is determined by the point with the smallest second derivative between point F and RV.	
	B/A, E/A, F/A, G/A, and H/A	|B/A|, |E/A|, |F/A|, |G/A| and |H/A|. Since B/A, F/A, and H/A were negative, we converted them to their absolute values for more intuitive interpretation. C/A and D/A were not used in this study due to difficulties in identifying points C and D under poor signal quality.	
	AI^w^	AI is typically defined as B/A – (C/A + D/A + E/A) [51]. However, in this study, AI is defined as the difference between B/A and E/A only, due to difficulties in identifying points C and D under poor signal quality, and because the values of C and D are close to zero.	
**Frequency-domain features**			
	PSDi^x^	The PSD function of the pulse waveform exhibits distinct peaks corresponding to fundamental sine‑wave components and their harmonics. Traditional approaches often quantify harmonic strength by peak heights; however, this metric is vulnerable to random noise. To address this, the strength of the i‑th harmonic (Sᵢ) is defined as the area under the PSD curve between the midpoint separating the ith and (i + 1)th peaks and the subsequent midpoint. Each midpoint is located halfway between 2 consecutive spectral peaks. This interval-based integration reduces the influence of noise on individual peak amplitudes and yields a more robust estimate of harmonic strength by effectively averaging the PSD over each frequency band (Figure 3). Since the is influenced by signal quality, the value of S_0_typically represents the average energy of the entire waveform, which is less relevant to the waveform contour. Additionally, the values of S_i_for i>6 are usually very small. Therefore, the energy strength of the i-th harmonic (PSD_i_) is normalized as: 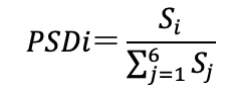 .	
	Vi	The weight of the i-th sinusoidal function that composes the waveform. Since any function can be decomposed into a Fourier series, Liu et al [39] proposed using the sum of sinusoidal functions to represent the waveform for further analysis. So, for each sample, f(t) can be decomposed as 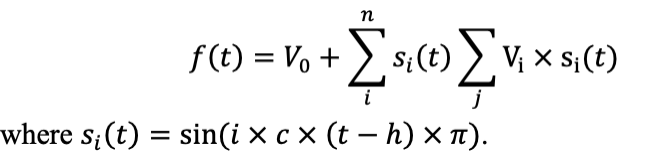 .	
	NHA^y^	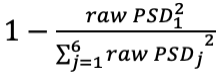 [52]	
	IHAR^z^	(1-NHA)/IPA [52]	

^a^SI: stiffness index.

^b^PPT: peak-to-peak time.

^c^ESP: early systolic peak.

^d^LP: left peak.

^e^FP: first peak.

^f^DP: diastolic peak.

^g^RV: right valley.

^h^BBI: beat-to-beat interval.

^i^LV: left valley.

^j^RI: reflection index.

^k^DPH: diastolic peak height.

^l^ESPH: estimated early systolic peak height.

^m^ERI: estimated reflection index.

^n^ESI: estimated SI.

^o^CT: crest time.

^p^NT: notch time.

^q^IP: inflection point.

^r^DP: dicrotic peak.

^s^DN: dicrotic notch.

^t^IPP: inflection point percentage.

^u^DT: diastolic time.

^v^IS: Inflection slope.

^w^AI: aging index.

^x^PSDi: the i-th relative power spectrum density.

^y^NHA: normalized harmonic area.

^z^IHAR: inflection and harmonic area ratio.

### Psychological Measures

To assess well-being, 3 self-report measures were administered: the Satisfaction With Life Scale (SWLS) [53], the Subjective Vitality Scale (SVS) [54], and the positive and negative affect schedule (PANAS) [55]. The SWLS comprises 5 items rated on a 7-point Likert scale ranging from “strongly disagree” to “strongly agree.” The SVS comprises 7 statements scored on a 7-point scale from “not at all true” to “very true.” The PANAS consists of 20 adjectives assessing positive and negative affect over the past week, with responses provided on a 5-point scale (1=“very slightly or not at all,” 5=“extremely”). Separate scores for positive affect (PANAS-P) and negative affect (PANAS-N) were computed to enhance interpretability.

Common mental health measures included the Patient Health Questionnaire-9 (PHQ-9) [56] and the Generalized Anxiety Disorder-7 (GAD-7) scale [57]. The PHQ-9 includes 9 items rated from 0 (“not at all”) to 3 (“nearly every day”), with total scores categorizing depressive symptom severity from minimal to severe. The GAD-7 comprises 7 items using the same response scale and classifies anxiety levels as normal, mild, moderate, or severe based on the summed score.

Momentary emotional states were captured using the Self-Assessment Manikin (SAM) [58], a nonverbal pictorial instrument representing affective valence and arousal. Participants selected the figure that best reflected their current level of pleasure (valence) and activation (arousal). The dominance dimension was omitted, as it was not directly relevant to the study’s objectives. All questionnaires were completed before signal collection.

### Data Analysis

Associations between individual pulse-waveform features and psychological measures were first examined using simple linear regression, which provides a transparent and interpretable baseline for evaluating feature–outcome relationships and facilitates comparison with prior psychophysiological studies relying on univariate analyses.

The data exhibited a nested structure: psychological scales were administered once per participant, whereas pulse-waveform features consisted of multiple repeated measurements per participant. Treating all observations as independent under standard ordinary least squares assumptions would therefore be inappropriate and could result in underestimated standard errors and biased statistical inference. To explicitly address this nonindependence, 2 complementary analytic strategies were implemented.

The first strategy involved participant-level aggregation, whereby waveform features were summarized for each individual using median values and were entered into ordinary least squares models. This approach mitigates within-subject dependence by aligning the unit of analysis for physiological features with that of the psychological measures. The second strategy applied cluster-robust standard errors at the original observation level, allowing statistical inference to account for repeated measurements within participants while preserving within-person variability.

From a theoretical perspective, participant-level aggregation may be more appropriate in this context, as cluster-robust standard errors can be less suitable when the dependent variable is constant across observations within the same cluster [59]. However, this aggregation approach necessarily entails a loss of within-participant information. Taken together, these 2 analytical strategies offer complementary protections against bias arising from nonindependent observations.

In addition, because multiple waveform features were tested following feature selection, statistical significance was evaluated using a Bonferroni-corrected threshold [60,61]. Although conservative, this correction was adopted to control the error rate, with the acknowledgment that it may exclude features of potential substantive relevance [62].

To evaluate predictive utility beyond univariate linear models, machine learning methods were further used to model psychological outcomes from multivariate pulse-waveform features. These methods are well-suited to this context because they can capture nonlinear relationships, complex feature interactions, and high-dimensional structures that may not be adequately modeled using traditional regression. Model performance was assessed using 5-fold cross-validation to obtain stable estimates of generalization performance while maintaining sufficient training data in each fold. Among the evaluated algorithms, random forest (RF) models were selected due to their robustness to multicollinearity, resistance to overfitting in moderate sample sizes, and ability to model nonlinear associations without strong parametric assumptions.

In each fold, the RF model was trained on data from a subset of participants and evaluated on held-out participants. Performance was quantified using out-of-sample metrics, including mean absolute error and the correlation between predicted and observed values, averaged across folds. The RF models were implemented using the default hyperparameters provided by scikit-learn. No manual hyperparameter tuning was performed. Specifically, the number of trees was set to 100, node splitting used the mean squared error criterion, trees were grown to full depth (no maximum depth specified), and bootstrap sampling was enabled. Other parameters remained at their library default values.

To prevent data leakage and preserve independence between training and evaluation, all observations from a given participant were assigned exclusively to either the training or test set. For all data analyses, an IQR multiplier of 1.5 was applied for outlier removal.

Agreement between predicted and observed values was further assessed using Bland–Altman analysis. Unlike correlation-based metrics, this approach evaluates absolute agreement and identifies potential systematic bias between predictions and ground-truth values, providing a complementary assessment of model validity that is particularly relevant for evaluating practical predictive performance.

For oximeter-photoplethysmography agreement analysis, 3 time-domain pulse waveform features commonly reported in prior studies, RI, FN, and diastolic time, were selected as representative indicators. Pearson correlation analyses were conducted to assess the linear association between the 2 measurement systems for each feature. Because correlation does not imply measurement agreement or interchangeability, Bland–Altman analysis was additionally performed to evaluate method agreement by plotting the differences between measurements against their means, thereby visualizing systematic bias and the 95% limits of agreement. The mean difference indicates directional bias, whereas the limits of agreement define the expected range of measurement differences.

Agreement was considered acceptable when most observations fell within the limits of agreement without systematic trends across the measurement range. Together, correlation and Bland–Altman analyses enabled a comprehensive evaluation of both association strength and measurement agreement between the smartphone-based photoplethysmography system and the clinical-grade fingertip oximeter.

### Ethical Considerations

The study protocol was approved by the Ethics Board of the Department of Psychology at Tsinghua University (number 201910). All study procedures were conducted in accordance with institutional and national ethical guidelines for human subjects research. Written informed consent was obtained from all participants prior to participation.Each participant received 50 renminbi (approximately US $7) as compensation for participation. Participants were informed about the study procedures and their right to withdraw at any time without penalty. All data were deidentified prior to analysis to protect participant privacy. Access to the original identifiable data were restricted to the first author, and no personally identifiable information was included in the analyses or reported results.

## Results

### Data Collection

Of the 127 participants recruited, 121 completed all questionnaires (71/121, 58.68% male; mean age 22.79, SD 1.87 years), and 113 of these participants completed the pulse-waveform recording (67/113, 59.29% male; mean age 22.74, SD 1.83 years). The 2 participants who exhibited severe depressive symptoms, as indicated by questionnaire assessments, were excluded at this stage.

**Figure 4:**
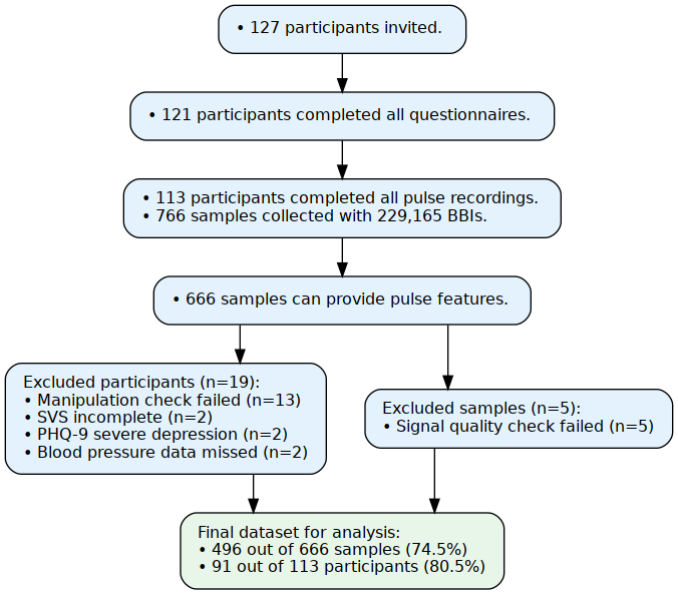
Participant and data filtering process. BBI: beat-to-beat interval; PHQ-9: Patient Health Questionnaire-9; SVS: Subjective Vitality Scale.

From the remaining 113 participants, a total of 766 waveform samples were collected (mean 6.78 samples per participant), comprising 229,165 BBIs (BBIs; mean 299 BBIs per sample). Of these, 100 samples were excluded due to partial feature loss (666 remaining; 86.9%), primarily resulting from poor signal quality that prevented reliable peak and valley detection (eg, HR could not be computed), although all recordings were successfully completed. Additionally, 19 participants were excluded because of problematic questionnaire responses identified through manipulation checks or severe depressive symptoms, and 5 samples with a signal quality index below 0.5, as proposed by Liu et al [39], were removed. After all exclusions, 496 valid samples from 91 participants were retained for analysis (Figure 4).

### Correlation Among Pulse Waveform Features

**Figure 5:**
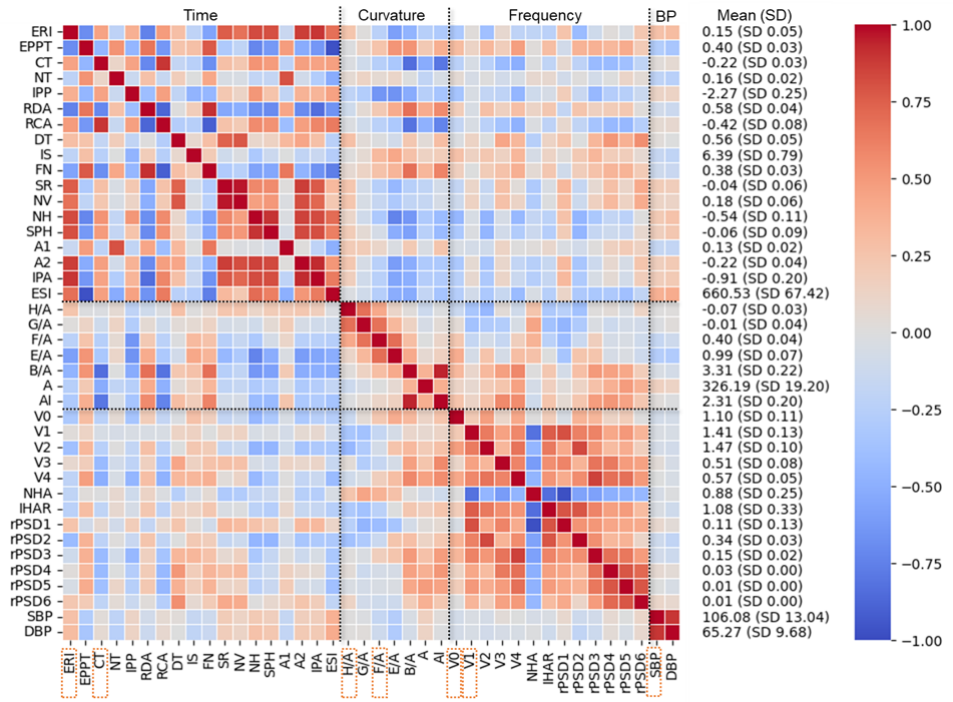
The correlation matrix of pulse waveform features. The orange boxes indicate the selected variables after correlation coefficient threshold filtering. DBP: diastolic blood pressure; DT: diastolic time; EPPT: estimated PPT; ERI: estimated reflection index; ESI: estimated SI; ESP: the early systolic peak; ESPH: early systolic peak height; IP: inflection point; IPA: inflection point area. (A2/A1); IPP: inflection point percentage; IS: inflection slope; NH: notch height; NT: notch time; NV: the interval between the second peak to right valley; PPT: peak-to-peak time; PSD: power spectrum density; RCA: the ratio of CT to NT; RDA: the ratio of NT to the combined duration of CT and DT; RI: reflection index; RV: right valley; SBP: systolic blood pressure; SI: stiffness index; SP: second peak; SPH: second peak height; SR: the interval between the second peak to right valley.

Features exhibited strong within-domain correlations across the time, curvature, and frequency domains, whereas cross-domain correlations were comparatively weak (Figure 5). To address high collinearity in the data, a systematic feature-selection procedure was applied prior to statistical modeling to reduce multicollinearity and improve model stability, parameter estimation, and inferential validity. Within each domain, feature pairs with absolute correlations exceeding a prespecified threshold of 0.5 were considered highly collinear and addressed through sequential elimination, after which the top 2 variables were retained for further analysis. Following this procedure, the final feature set used in subsequent analyses comprised estimated reflection index, crest time (CT), the third curvature minimum (F/A), the fourth curvature minimum (H/A), the first power spectrum density component (rPSD1), the baseline of Fourier decomposition (V0), and systolic blood pressure.

### Linear Regression and Correlation Analysis

**Figure 6:**
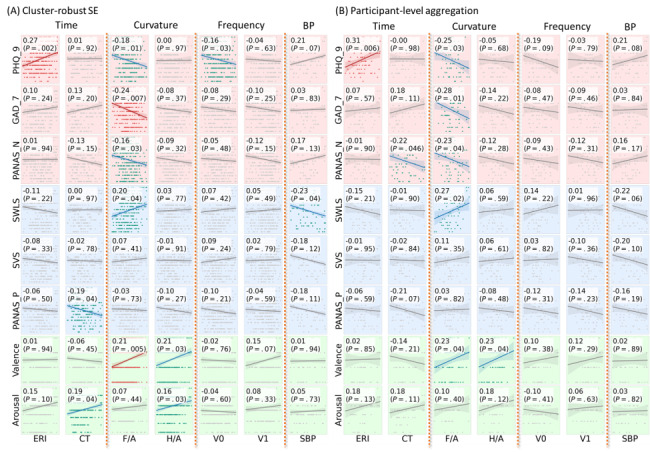
Univariate relationships between each independent and dependent variable. Green markers and regression lines denote *P* value less than .05 and red markers denote *P* value less than the Bonferroni corrected criteria .05/9=.0056. CT: crest time; ERI: estimated reflection index; GAD-7: Generalized Anxiety Disorder-7; PANAS: positive and negative affect schedule; PANAS-N: negative affect; PANAS-P: positive affect; PHQ-9: Patient Health Questionnaire-9; SBP: systolic blood pressure; SVS: Subjective Vitality Scale; SWLS: Satisfaction with Life Scale.

In univariate regression and correlation analyses, we examined associations among 8 psychological measures, 9 pulse waveform features, and blood pressure. The results indicated that negative psychological states were primarily associated with time- and curvature-domain waveform features (Figure 6). Depressive symptoms (PHQ-9) were significantly associated with F/A and V0, with the association between PHQ-9 and estimated reflection index remaining significant after Bonferroni correction. Generalized anxiety (GAD-7) also showed a Bonferroni-corrected significant association with F/A. Negative affect (PANAS-N) was associated with both CT and F/A.

In contrast, positive affect exhibited fewer significant associations with waveform features: SWLS was correlated with F/A, and PANAS-P was correlated with CT. Regarding momentary emotional states, valence was associated with F/A and H/A, whereas arousal was associated with CT and H/A.

### Machine Learning

**Figure 7:**
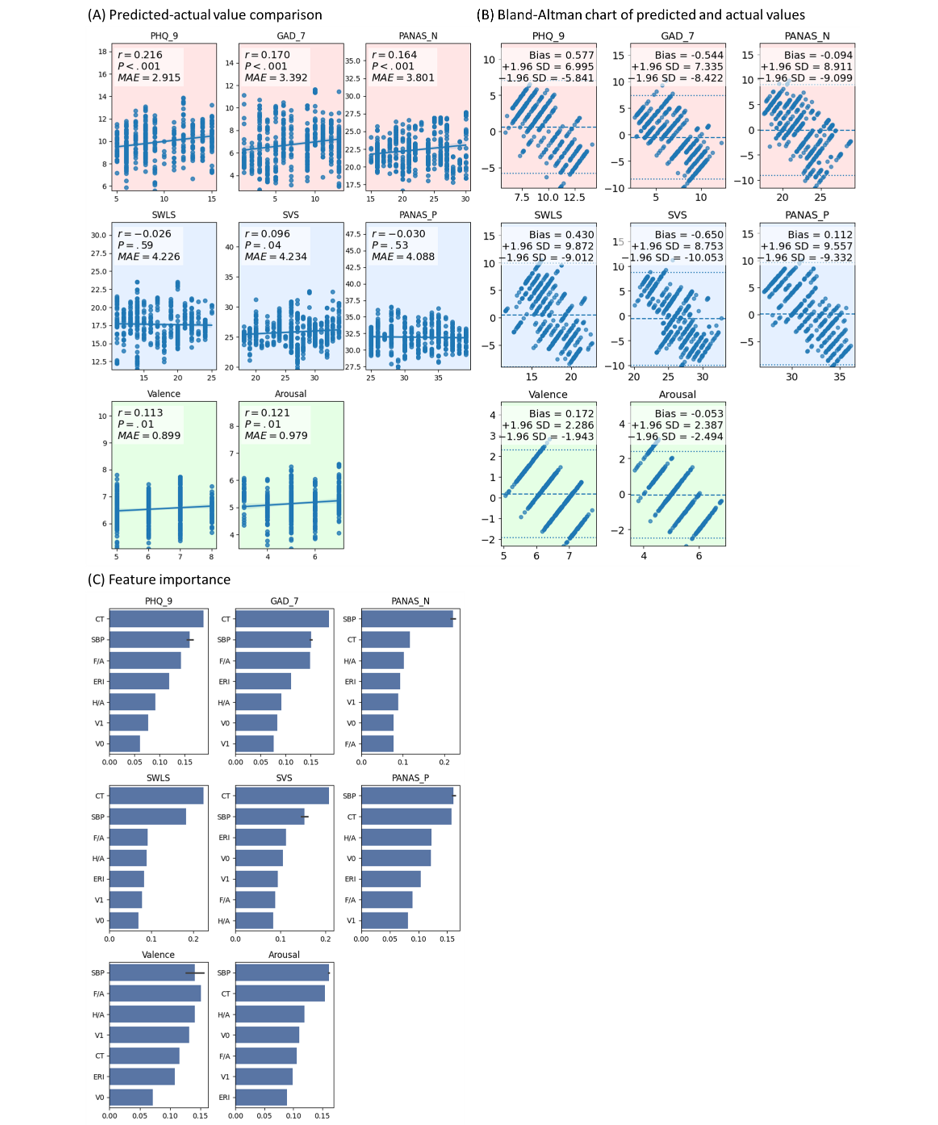
Machine learning results. GAD-7: Generalized Anxiety Disorder-7; MAE: mean absolute error; PANAS: positive and negative affect schedule; PANAS-N: negative affect; PANAS-P: positive affect; PHQ-9: Patient Health Questionnaire-9; SVS: Subjective Vitality Scale; SWLS: Satisfaction with Life Scale.

In this study, 9 features selected through feature engineering were used as inputs to predict mental health outcomes. The machine learning results (Figure 7) indicated that waveform-related features were effective in predicting mental disorders (*P*<.001 for PHQ-9, GAD-7, and PANAS-N), whereas predictive performance for positive emotional states was substantially weaker. With respect to short-term emotional fluctuations, heartbeat waveform features showed no clear association with valence but were evidently influenced by arousal. Bland–Altman analysis further demonstrated that, for outcomes with significant predictive correlations, the machine learning predictions exhibited slight but not substantial systematic bias. The 3 most important features identified by the RF models across all dependent variables were CT, F/A, and systolic blood pressure.

### Oximeter Comparison

**Figure 8:**
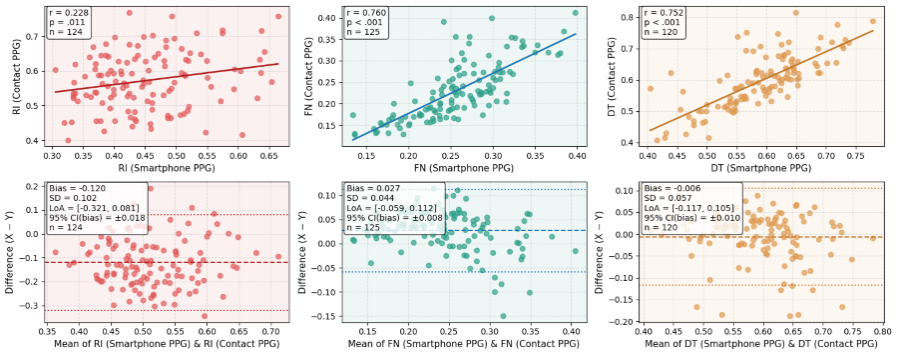
The agreement between the proposed smartphone photoplethysmography-based pulse waveform features and those obtained by oximeter. DT: diastolic time; FN: the interval between first peak to notch; PPG: photoplethysmography; RI: reflection index.

The results (Figure 8) indicate reasonable agreement between waveform features obtained from the oximeter and those derived from the smartphone-based photoplethysmography method. For all 3 features analyzed, correlation coefficients were statistically significant (*P*<.05), and Bland–Altman analyses revealed minimal systematic bias. However, consistent with the optical boundary effects inherent to smartphone photoplethysmography acquisition discussed in the waveform metrics section, the reflection index exhibited some degree of distortion. In contrast, time-related waveform features were largely unaffected by these optical limitations and showed more consistent agreement across the 2 measurement modalities.

## Discussion

### Principal Findings

Overall, the findings support the study’s hypotheses and objectives by demonstrating that fingertip pulse-waveform signals recorded using a standard smartphone can be used to predict individual psychological health status. This work makes 3 principal contributions.

First, we introduce and validate a cost-effective, noninvasive approach that relies solely on a smartphone camera to capture subtle skin color variations, reconstruct digital arterial pulse waveforms, and infer mental health status. Across both univariate linear regression and multivariate machine learning analyses, waveform features showed consistent and statistically significant associations with psychological indicators. Beyond extending smartphone-based waveform analysis to psychological research, these findings provide additional empirical support for pulse-waveform–based psychological assessment more broadly [34,35], as well as for the use of smartphones in related physiological applications such as HRV and blood pressure analysis [63,64].

Second, our results reveal associations between pulse-waveform features and psychological variables across multiple feature domains. Consistent with findings reported by Kontaxis et al [34] and Celka et al [65], negative psychological conditions were strongly linked to waveform characteristics, suggesting that long-term psychological states may induce structural or functional changes in the arterial system. However, contrary to our initial expectations, these associations were primarily observed in curvature- and time-domain features rather than in the frequency domain. As one of the first studies to incorporate frequency-domain pulse-waveform features into mental health research, we suggest that such features may encode relevant information, although this was not supported by the present dataset.

The relatively weaker associations observed between positive affect and pulse-waveform features were also unexpected but appear reasonable in retrospect. Given the well-documented negative correlation between positive and negative affect and evidence that positive affect plays a protective role against adverse psychological states [66], we anticipated that positive affect would exhibit associations with waveform features comparable to those observed for negative affect. Although the observed relationships were directionally consistent with this hypothesis, they were generally weaker and less statistically robust. This pattern remains theoretically consistent with prior psychological research, which emphasizes that positive and negative affect are distinct, partially independent constructs that can co-occur within individuals despite their negative correlation [67]. Together, these findings suggest that both affective dimensions relate to cardiovascular functioning, but that negative affect exerts a more pronounced influence.

Short-term emotional fluctuations appear to exert limited influence on vascular wall properties. However, because arousal is closely linked to HRV [68], which directly affects the timing of cardiac contractions, it is reasonable that arousal was associated primarily with time-domain waveform features. This interpretation aligns with earlier work in affective computing by Healey and Picard [69], who demonstrated that physiological signals reliably capture arousal but are less effective at distinguishing affective valence. Although the present findings are supported by emerging evidence, further studies using higher-quality data and more controlled experimental designs are needed to confirm and extend these results.

### Limitations and Future Directions

Despite the theoretical and practical innovations of this study, 3 limitations warrant cautious interpretation of the results.

First, although the machine learning models achieved significant predictive performance, the level of agreement between the proposed method and the target psychological measures remained limited. Similarly, comparisons between waveform features derived from the smartphone-based photoplethysmography method and those obtained from a reference oximeter demonstrated statistically significant associations, but the magnitude of agreement was insufficient for precise interchangeability. Taken together, these findings indicate that, while pulse waveform features are systematically influenced by mental health status, the current models primarily capture correlational relationships rather than yielding predictions with practical accuracy.

Second, of the 766 waveform samples collected, 100 (13.1%) were classified as corrupted and excluded, a substantial loss rate that may undermine the study’s validity. In uncontrolled, real-world settings, slight finger movement or fluctuations in ambient illumination can distort the photoplethysmography signals [70]. Future research can therefore use enhanced image capture protocols [71], integrate advanced signal processing and noise reduction algorithms [72], and implement quality filters to discard low-fidelity recordings [73]. Moreover, the limited and variable frame rates of smartphone cameras (typically 10-60 Hz) constrain temporal resolution compared with medical-grade devices sampling up to 1000 Hz [74]. Because smartphone camera frame rates are not fixed, this instability further exacerbates data-collection challenges [75]. The effects of reduced and unstable sampling rates on photoplethysmography signal integrity remain poorly understood and warrant further investigation [76].

Third, the sample consisted primarily of young adults with similar educational backgrounds, which may limit the generalizability of the findings. Because vascular changes associated with psychological factors often develop over time, associations between mental health and pulse-waveform morphology may be more pronounced in older adults or individuals with chronic conditions. Future studies should therefore include participants across broader age ranges and with more diverse health profiles. Additionally, the slight gender imbalance in the sample may have introduced bias in sex-specific waveform effects. Other potential confounders, such as sleep quality, smoking, diet, and medication use, were not controlled for and should be addressed in future research to reduce unexplained variance.

### Conclusions

Pulse waveform analysis has traditionally been applied to the assessment of physical health, whereas its extension to mental health, via links between psychological states and vascular stiffness, remains relatively novel. Conventional waveform acquisition methods are often costly and inaccessible, which has limited their adoption in psychological research. This study uses smartphone-based photoplethysmography to analyze digital arterial waveforms, thereby providing a noninvasive and accessible approach for psychological assessment. The findings demonstrate significant associations between psychological variables and pulse-waveform features, particularly within the time- and curvature-domains, and thus contribute to the advancement of psychophysiological research grounded in hemodynamic principles. Nevertheless, technical limitations (eg, signal variability and low, unstable sampling rates) and sample-related constraints (eg, demographic homogeneity and uncontrolled confounders) should be addressed in future studies.

## Abbreviations

BBI: beat-to-beat interval
CT: crest time
GAD-7: Generalized Anxiety Disorder-7
HR: heart rate
HRV: heart rate variability
PANAS-N: negative affect
PANAS-P: positive affect
PANAS: positive and negative affect schedule
PHQ-9: Patient Health Questionnaire-9
RF: random forest
SAM: Self-Assessment Manikin
SVS: Subjective Vitality Scale
SWLS: Satisfaction With Life Scale

## Data Availability

The datasets generated or analyzed during this study are available in the GitHub repository [77].
